# Why is the screening rate in lung cancer still low? A seven-country analysis of the factors affecting adoption

**DOI:** 10.3389/fpubh.2023.1264342

**Published:** 2023-11-09

**Authors:** Charlotte Poon, Tim Wilsdon, Iqra Sarwar, Alexander Roediger, Megan Yuan

**Affiliations:** ^1^Charles River Associates, London, United Kingdom; ^2^MSD International Business GmbH, Kriens, Switzerland; ^3^Merck & Co., Inc., Kenilworth, NJ, United States

**Keywords:** lung cancer, screening, early detection, adoption, country analysis

## Abstract

Strong evidence of lung cancer screening’s effectiveness in mortality reduction, as demonstrated in the National Lung Screening Trial (NLST) in the US and the Dutch–Belgian Randomized Lung Cancer Screening Trial (NELSON), has prompted countries to implement formal lung cancer screening programs. However, adoption rates remain largely low. This study aims to understand how lung cancer screening programs are currently performing. It also identifies the barriers and enablers contributing to adoption of lung cancer screening across 10 case study countries: Canada, China, Croatia, Japan, Poland, South Korea and the United States. Adoption rates vary significantly across studied countries. We find five main factors impacting adoption: (1) political prioritization of lung cancer (2) financial incentives/cost sharing and hidden ancillary costs (3) infrastructure to support provision of screening services (4) awareness around lung cancer screening and risk factors and (5) cultural views and stigma around lung cancer. Although these factors have application across the countries, the weighting of each factor on driving or hindering adoption varies by country. The five areas set out by this research should be factored into policy making and implementation to maximize effectiveness and outreach of lung cancer screening programs.

## 1. Introduction

Cancer was responsible for almost 10 million deaths in 2020 and is one of the leading causes of deaths globally (1). Lung cancer accounts for the highest number of cancer deaths; it is responsible for almost 20% of cancer deaths (1). Approximately 75% of lung cancer patients diagnosed are in a late stage, resulting in a poor prognosis (2). The Global Cancer Observatory suggests that the net one-year survival rate decreases as the stage of disease progresses (80.0–93.4% for localized disease vs. 19.4–25.7% for distant lung cancer) (3).

The World Health Organization (WHO) has identified two main approaches to improve early detection of cancer: early diagnosis and screening. The WHO defines early diagnosis as “awareness of early signs and symptoms of cancer to facilitate timely diagnosis before the disease progresses to advanced, in order to allow effective treatment.” Screening is the “application of a screening test in a population presumed to be asymptomatic to identify those that may have suggestively cancerous abnormalities.” The WHO finds screening programs effective when they are applied to over 70% of the at-risk population, provided that all the necessary infrastructure and resources are in place (4). The European Union (EU) Council Recommendations published in September 2022, which have been adopted by EU health ministers and were signed by all member states in December 2022, support early detection of cancer and in particular point out the need for countries to explore the feasibility and effectiveness of a lung cancer screening program coupled with smoking cessation interventions (5). In May 2022, as part of the Cancer Moonshot Initiative in the United States (US), President Biden called for action on cancer screening (6), including development of person-centered and sustainable approaches to bringing cancer screening to communities, especially rural America; mobile screening; and specific funds for cancer screening. Other initiatives focus on expanding messaging and supporting early detection by bringing together leading organizations for national roundtables on improving screening and leveraging social media campaigns. While in European and United States, 70% of lung cancer patients are ever smokers, there is a high incidence of lung cancer in never smokers in Asia which means targeted strategies are needed to ensure the right sub-population is screened (7).

At the national level, some governments decided to implement formal screening programs for lung cancer. A study conducted by Poon et al. identified a number of factors influencing policy makers’ decisions to implement formal screening programs: (1) recognition of the disease burden and the value of early detection, (2) strong clinical data showing mortality reduction and benefit–risk analysis relevant to the local context, (3) cost-effectiveness data and budget impact, (4) local feasibility demonstration, and (5) a clear and integrated decision-making mechanism involving relevant stakeholders (8). A few countries have made strides in implementing formal lung cancer screening programs, including the US, the first country to support screening. Data suggest that only 5.8% of the 14.2 million people eligible for screening in the US have been screened (the latest data is from 2021). Low uptake is observed in other countries that have implemented formal screening programs, namely Japan, South Korea, Croatia, and Canada. This raises key questions about the underutilization of lung cancer screening and highlights that action is needed to ensure widespread participation and long-term sustainability of the programs to prevent cancer-related deaths (8).

In this study, we seek to understand how lung cancer screening programs currently perform and identify enablers and barriers affecting adoption while considering WHO’s recommendations about program design. We hope this paper will inform policy making, policy advocacy, and program design.

## 2. Methods

### 2.1. Manuscript formatting

This is a case study analysis that reports on common factors cited as having an impact on the adoption of lung cancer screening. The review does not cover the specific design of a lung cancer screening program impacting efficiency and quality of screening but the general factors which affect participation in screening.

**Figure 1:**
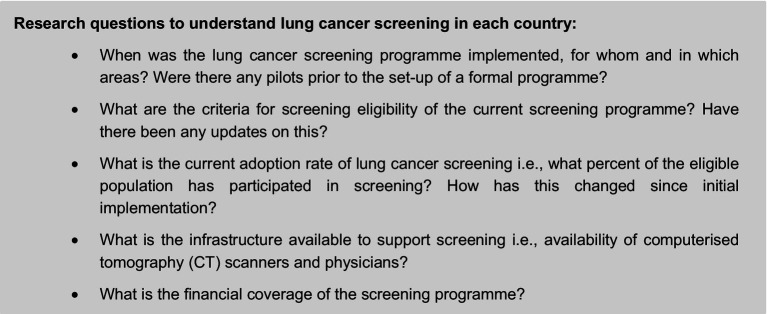
Set of research questions. Source: CRA analysis.

First, we conducted a review of the literature on the background and performance of the screening program and factors affecting adoption, with adoption defined herein as participation in formal lung cancer screening program. The review includes lessons drawn from the pilot programs in each country within the scope of our study—Japan, US, South Korea, China, Croatia, Canada, and Poland—to answer a set of research questions (see Figure 1). These countries were selected based on two criteria: (1) a formal lung cancer screening program already exists at the point of initiating this research in May 2022, and (2) there is national/federal recommendation, or at least *a funding commitment*, to implement a program.

Search terms used on PubMed to identify the potential factors were “adoption of lung cancer screening,” “success of lung cancer screening,” “uptake,” and “lessons learnt pilot programs.” Where feasible, English reports were studied, but local language searches were conducted to ensure comprehensiveness. A total of 91 unique articles published in the last 5 years were reviewed. They include academic articles about lung cancer and other cancer screening; governmental official reports, i.e., National Cancer Plans; lung cancer strategies; and nongovernmental organisation (NGO) publications from lung cancer patient advocacy groups, clinical societies, and media reports.

Finally, we distilled the key factors that affect the adoption of lung cancer screening. A qualitative approach was used where the frequency of a factor cited in literature was identified. The number of “occurrences” of the factor was a proxy for the importance of the factor in driving adoption. We validated our analysis of factors affecting the adoption of lung cancer screening in the scope countries using a set of interviews, conducted through 1 h teleconference. The set of factors was validated with three key stakeholders representing perspectives from the policy, payers, and patient representative angles, namely, with Dr. Christine Berg, former chief of the early detection research group at the National Cancer Institute (US), Mr. Ivica Belina, President of The Coalition of Associations in Healthcare (Croatia) and Dr. Yeol Kim, Head of the Division of Cancer Management Policy at the National Cancel Center (South Korea). Each stakeholder also provided regional expertise from North America, Europe, and Asia.

## 3. Review of the barriers and enablers and implications

We collate the evidence of barriers and enablers in the adoption of lung cancer screening programs across seven case study countries: Canada, China, Croatia, Japan, Poland, South Korea, and the US.

Given the varying times when screening was introduced, there is inevitably more evidence in some markets than others. For example, many studies have been done on lung cancer screening in the US from 2000 to 2007, with results published in 2010, but less public information is available on the Croatian national screening program, which took off only in October 2020 and experienced significant delays because of disruptions stemming from the coronavirus disease (COVID-19) pandemic (9). Therefore, we also included lessons learned from screening of other major tumor types, such as breast cancer and cervical cancer, in these countries and then considered the extent to which they are likely to apply to national lung cancer programs.

The case studies have been presented in chronological order based on when a national lung cancer screening program was implemented in the country. Each case study begins with evidence that is available on the adoption of lung cancer screening, followed by conclusions inferred from proxy data. A summary of answers to the research questions asked about lung cancer screening programs across countries can be found in Table 1.

**Table 1:** Indicators for lung cancer screening.

Country	Introduction of screening		Screening eligibility criteria	Total number of individuals eligible for screening	Adoption rate (most recent data)	Infrastructure		Insurance coverage
	Pilots	Formal program implementation				CT scanners (per 1 million population)*	Physicians (per 100,000 population)	
Japan	–	In place for decades; National Cancer Centre published guidelines recommending chest X-rays and sputum cytology in 2006	Men and women aged 40–79 years	~3 million (based on a simulation model for any year between 2020 and 2040)	53.4% (men) (2019) 45.6% (women) (2019)	171.3 (CT, MRI, and PET scanners)	250	Diagnostic tests are covered by national health insurance
US	LSS (2000) NLST (2002–2007)	Centers for Medicare and Medicaid services began covering LCS in 2015 under prescription from a physician	50–80 years of age with a 20 pack-year smoking history	14.2 million (4.83% of population based on 2021 figures**)	5.8% in 2021	43	260	CT scans cost $300 and are usually covered by insurance. However, for those without insurance or Medicaid, this can result in high OOP costs
South Korea	K-LUCAS (2017)	The NLCSP was implemented in 2019 managed by the KNCSP	Current smokers aged 54–74 years with a 30 pack-year smoking history	3.6 million (6.9% of population based on 2021 figures**)	23% underwent screening in 2019 and 2020	38.2	250	The NHIS covers 90% of the cost of KNCSP. LDCT costs approximately US$100, of which patients pay 10%, while the full cost is covered or the lower 50% income group
China	People’s Republic of China National Cancer Early Screening Trial (2019)	RuraCSP began in 2010 across six provinces for high-risk individuals living rurally CanSPUC began in 2012 for those in the general community population	National Cancer Early Screening Trial eligibility is those aged 50–74 years with a 20 pack-year smoking history who are current smokers or quit in the past 5 years	~30 million (based on a simulation model for year 2025)	National participation rate across China sits at 6–31%	11.24 (highest number across 5 provinces)	220	LDCT is approximately US$80 and is covered by health insurance
Croatia	–	Nationwide screening program implemented in 2020	Active smokers aged 50–70 years and those who quit smoking in the past 15 years	n/a	10,000 people have been screened as of December 2022	17 CT scanners across the whole country	300	–
Canada	Ontario pilot (2017) Alberta study (2015) Quebec demonstration project (2021)	British Columbia implemented a formal program in 2022	55–74 years of age who are currently or have previously smoked and have a 20 pack-year smoking history	n/a	–	14.6	242 (Quebec) 237 (Alberta) 196 (Saskatchewan)	Other cancer screenings (such as breast cancer) are free of charge in Quebec if undertaken at a designated screening center
Poland	Szczecin, Gdansk, Poznan, Warsaw pilot studies (2008) National pilot (2020)	Formal national program to be set up in 2023	Active smokers aged 55–74 years and those who quit smoking in the past 15 years with a 20 pack-year smoking history	n/a	–	18.2	240	Healthcare is free for all citizens through the publicly funded healthcare system

Sources: multiple sources listed in country sections.

*The OECD dataset has been selected for benchmarking because of its comprehensiveness, which allows comparison across countries.

**The percentage is calculated based on data taken from The World Bank.

### 3.1. Japan

#### 3.1.1. Background

There has been population-based screening, i.e., screening offered systematically to everyone in the defined target group (men and women aged 40–79 years) using chest X-ray and sputum cytology for over two decades (10). Despite randomized controlled trials in the US and Europe suggesting that chest radiography is not an effective intervention and that low-dose computerized tomography (LDCT) is effective in reducing mortality, Japan has maintained its recommendation for X-rays and sputum cytology. This appears to be based on multiple case–control studies conducted in Japan that have shown that chest radiography and sputum cytology reduce lung cancer mortality (11). In 2006 the National Cancer Centre (NCC) published guidelines laying out the eligibility criteria for lung cancer screening. A simulation model based on any year between 2020 and 2040 suggests that approximately 3 million people in Japan are eligible to receive screening (12). While guidelines for implementation have been established, no government targets for screening have been set out yet.

#### 3.1.2. Adoption

The rate of lung cancer screening has improved over the years. In 2010, 26.4% of men and 23% of women in the eligible population were reported by the National Cancer Centre to have been screened, vs. 53.4 and 45.6%, respectively, in 2019 (13).

#### 3.1.3. Factors affecting adoption

We found only one study focusing on factors affecting adoption. A 2016 Public Opinion Survey on cancer control showed that the public fails to see the benefits of early detection in lung cancer. Respondents cited confidence in their own health, a lack of time, and easy access to hospitals if and when they begin to worry about their health as the key reasons for nonparticipation in screening (14). There is a need to improve disease awareness and cancer literacy and to clearly define the role of primary care physicians in lung cancer care. Better-organized dissemination of guidance on who is eligible for screening and when they are eligible is needed.

Financial considerations do not appear to be a barrier to adoption. Many diagnostic imaging tests in Japan are covered by national health insurance, so accessibility is high (11). As for healthcare infrastructure, Japan has the highest number of CT, magnetic resonance imaging (MRI), and positron emission tomography (PET) scanners among all Organisation for Economic Co-operation and Development (OECD) countries: 171.3 per 1 million population, almost four times the OECD average (15). According to 2019 OECD data, there are 250 physicians per 100,000 population in Japan, which is lower than the OECD average of 360 per 100,000 population (15).

#### 3.1.4. Other screening programs

More generally, across cancer screening, the literature focuses on the role of education. A 2022 study looking at cancer screening rates finds that the percentage of screening participants with university or graduate school education was higher than the percentage with junior or senior high school education (16). This concludes that there is a relationship between higher education and a person’s desire for information about and awareness of their health status. The literature also considers the effectiveness of different forms of communication. Each municipality sends invitations to those who are eligible for cancer screening, but this is not seen as effective in communities because the recipients lack information on cancer outcomes and the benefits of early detection (16). The study also concluded that having a family member who experienced cancer was associated with a better understanding of cancer and promoted an intention to undergo cancer screening rather than avoid it, particularly in regard to cancers with a genetic component (16).

There is also evidence of people’s attitude to screening. Psychological distress, including fear and anxiety about cancer, is linked to avoidance of cancer screening. A survey of Japanese students suggested that the number of people willing to undergo cancer screening when they were old enough to do so decreased as the school year progressed, implying that people are more likely to shun cancer testing when they are more likely to have it (16). There is also fear of potential harm from cancer screening—not only pain, but also the risk of radiation exposure. Anti-screening messages most commonly cite the risk of medical radiation exposure, suggesting it is several times higher than the risk of radiation exposure due to the atomic bombs dropped on Nagasaki and Hiroshima was (17, 18).

### 3.2. United States

#### 3.2.1. Background

**Figure 2:**
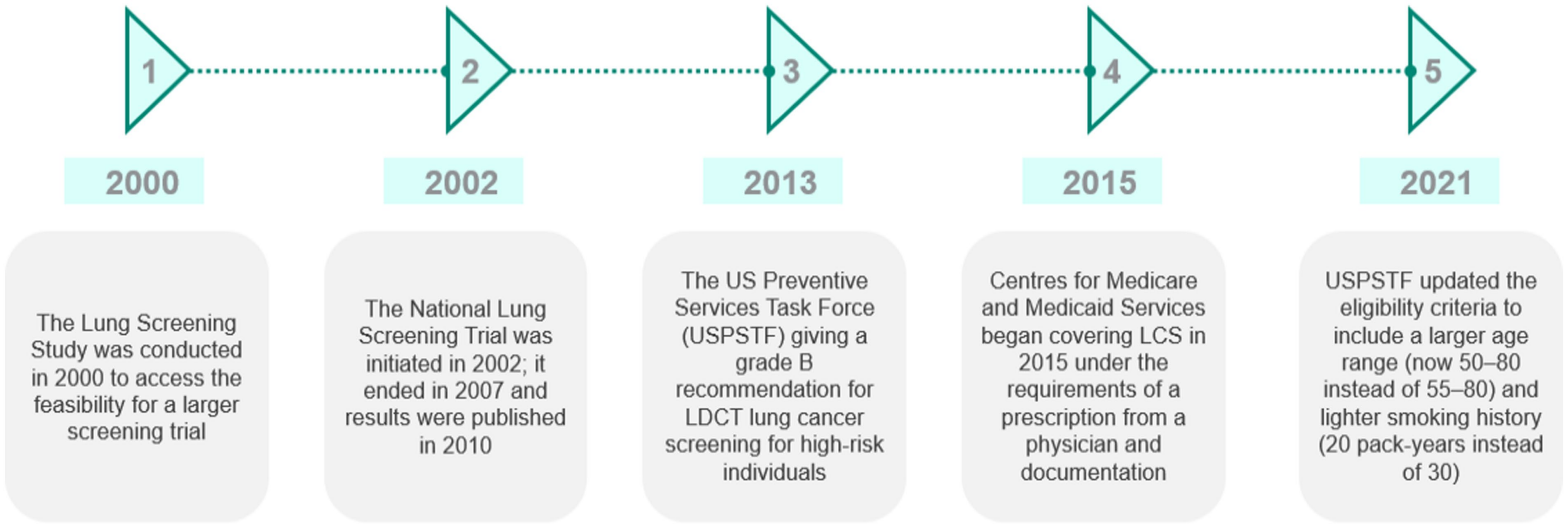
Summary of the key events and timeline in the development of lung cancer screening in the US. Source: CRA analysis.

The US became the first country to adopt lung cancer screening using low-dose CT in 2013. The Centers for Medicare & Medicaid Services began covering lung cancer screening in 2015 under the requirements of a prescription from a physician and documentation showing mutual decision-making of patients and physicians (19). In 2021, the United States Preventive Services Task Force (USPSTF) updated the eligibility criteria to include a wider age range (50–80 instead of 55–80) and lighter smoking history (20 pack-years instead of 30) (20). Data suggest that approximately 14.2 million people are eligible for screening (21). While no federal target for lung cancer screening has been set, given the fragmented healthcare service in the US, the US Department of Health and Human Services set a Healthy People 2030 target to increase the proportion of adults who receive screening to 7.5% (based on 2013 USPSTF eligibility guidelines). In 2021, 5.8% were screened (22). It is, however, important to note that this figure may underrepresent the proportion of the population screened as managed care providers and others such as the Veterans Administration do not report screening numbers to the ACR registry (23). A summary of the key timelines and events is illustrated in Figure 2.

#### 3.2.2. Adoption

Implementation has been slow. A study reviewing the 2015 National Health Interview Survey showed only a small increase in CT scan use for LCS from 2001 to 2015 (24). In 2015, just 4.4% of people who met the USPSTF criteria were screened. More recent data from 2019 show that only 5.8% of those who meet eligibility criteria have undergone screening (22).

#### 3.2.3. Factors affecting adoption

The existing literature identifies coverage and financial considerations as being important to screening adoption. It is estimated that half the population eligible for lung cancer screening have either no insurance or Medicaid, which varies by state, preventing uptake due to high out-of-pocket (OOP) costs (25). Beyond the direct cost, low-income populations face additional challenges in the form of ancillary costs, which can include the costs of transport to screening centers and taking sick leave to attend appointments.

What compounds the financial challenge is the fragmentation of the insurance system. The American Lung Association reported that eligibility criteria and use of prior authorization for lung cancer screening differ across managed care organizations (organizations or health plans focused on managed care as a model to limit costs while maintaining the quality of care). This fact may lead to poor continuity of care as patients move across providers and insurers (26).

Regarding health infrastructure, OECD data from 2021 show that in the US there were 43 CT scanners per 1 million population, higher than the OECD average of 26 (15). Data from 2019 show there were 260 physicians per 100,000 population, lower than the OECD average of 360 physicians per 100,000 population (27). Resources are not spread evenly across the country. An Association of American Medical Colleges registry shows that the northeast has the highest ratio of physicians per 100,000 population and the highest number of certified screening facilities, correlating with a higher uptake rate for the lung cancer screening program (28). A randomized controlled trial to engage underserved populations found that health systems with a screening clinic, full-time program coordinator, and free screenings had higher rates of lung cancer screening (29). While there is no evidence that the overall infrastructure is a barrier, there are challenges with the collection of data and entry into standard electronic medical records that limit appropriate identification of high-risk individuals, i.e., heavy smokers and those with a history of smoking (23).

In addition, physician awareness and communication of the benefits and risks of lung cancer screening seem suboptimal. A qualitative study investigating eligible participants’ reasons for opting out of screening found that participants felt that screening invitations from their general practitioner (GP) seem opportunistic as there was no discussion after the procedure (30). A 2017 study of US physician readiness in the implementation of programs found that only 42% of healthcare professionals (HCPs) were able to accurately identify eligible high-risk patients; this was attributed to a lack of knowledge regarding screening guidelines (31). However, physician awareness may have improved following the release of the Dutch–Belgian lung cancer screening trial (NELSON) data, because the American Academy of Family Physicians has supported education campaigns (23).

Patient-level barriers also play a role in uptake. A review of factors affecting cancer screening uptake among patients reported that stigma related to lung cancer being perceived as a self-inflicted disease and the fear of being blamed for a cancer diagnosis due to tobacco consumption has been identified as a factor making smokers less willing to consider lung cancer screening (32).

### 3.3. South Korea

#### 3.3.1. Background

The Korean Lung Cancer Screening demonstration project (K-LUCAS) was a nationwide pilot project that began in 2017 (33, 34). The successful completion of K-LUCAS led to the implementation in 2019 of the National Lung Cancer Screening Program (NLCSP) managed by the Korean National Cancer Screening Program (KNCSP), which already supported cancer screening for stomach, colon, breast, cervix, and liver cancer (33). Eligibility criteria include current smokers aged 54–74 years with a 30-pack-year history of smoking, and screening is be carried out every 2 years (33). It is suggested that around 3.6 million individuals are eligible for screening—6.9% of the South Korean population in 2021 (35). Invitation letters were sent to eligible high-risk people selected from an electronic database based on the survey carried out by the national health screening program. The government has set no identifiable screening targets (36).

#### 3.3.2. Adoption

Although the pilot program was deemed a success, the screening rate was lower than expected in comparison to other cancer screening programs, even after taking into account the impact of the COVID-19 pandemic (37). In 2019 and 2020, around 23% of the 690,000 eligible subjects underwent screening with LDCT (38). The most recent data show an increase in uptake, with a participation rate of 38% among eligible candidates in 2021 (39).

#### 3.3.3. Factors affecting adoption

Political support is seen to be an important facilitator in the success of lung cancer screening (LCS) programs. In South Korea, this can be seen through the heavy subsidization of LDCT as well as the invitation to screening being spearheaded by the National Health Service proactively reaching out to high-risk patients rather than relying on primary care physicians to refer patients when they deem it appropriate. The support of academics, such as radiologists and pulmonologists, and particularly the National Cancer Center has been suggested to be key to the success of the program (39).

The National Health Insurance Service (NHIS) in South Korea covers 90% of the cost of KNCSP. The cost of LDCT is approximately US $100, of which patients are required to pay only 10% (and it is free for those in the lower 50% income group) (40). The government subsidizing a large portion of the cost of LDCT has significantly improved affordability for patients. However, the cost of potential follow-up diagnostic tests and treatment can act as a deterrent for those in the lower income group (39). What is worth noting is that lower-income households had a strong preference for accessibility of the screening units over quality (accessibility being defined as distance to travel from home and quality being defined as being in a large referral hospital rather than a small hospital) (33). By contrast, people with a higher education level prefer quality over accessibility.

The National Cancer Center Lung Cancer Screening Quality Control Center found that many who received the invitation letter did not have a good understanding of the benefits of lung cancer screening and therefore rejected the offer (41). In the last NCC survey from 2018, the lack of knowledge about lung cancer symptoms, presentation, and risks was evident. High-risk patients in the country seem to be overconfident about their health and name “lack of time” as a reason to avoid participation. This is particularly dangerous because South Korea has the second-highest rate of smokers globally (behind Eastern Europe) (42). This is worsened by the public’s perception of the low accuracy of screening and fear of radiation exposure (43). Interestingly, men are more likely to have a negative view of screening, according to a public opinion survey on the National Lung Cancer Screening Program conducted in 2018 (33).

Regarding infrastructure, a 2018 survey suggests that there is widespread distribution of CT scanners with radiology specialists, meaning that even small to medium-sized hospitals are able to operate screening programs. According to OECD health data from 2019, there are 38.2 CT scanners per 1 million people as of 2017 (10.4% higher than the OCED average), and there were 250 physicians per 100,000 population (15, 33). However, challenges in accessing the services may remain. As mentioned, lower-income households had a strong preference for accessibility of the screening units over quality (accessibility defined as distance to travel from home and quality defined as large referral hospital vs. small hospitals), which suggests that shorter distances to travel for screening is a strong motivator for those of lower socioeconomic status (33). People with more education prefer quality over accessibility, as compared with those with less than a middle school education, for whom accessibility is a priority.

In a 2013 survey, only 17.3% of the physician respondents were aware of the findings from the NLST study in the US, and the majority of respondents believed that chest X-ray scans and LDCT were both effective as screening modalities (44). Since the implementation of the National Lung Cancer Screening Program in 2019, knowledge about LCS among both primary care physicians and radiologists has increased significantly (39).

### 3.4. China

#### 3.4.1. Background

Two large-scale, population-based, lung cancer screening programs have been organized in China: the Rural People’s Republic of China Screening Programme (RuraCSP), which began in 2010 across six provinces for high-risk individuals living rurally, and the Cancer Screening Program in the Urban People’s Republic of China (CanSPUC) Programme initiated in 2012.

In 2019, the first population-based randomized controlled trial for cancer screening began: the People’s Republic of China National Cancer Early Screening Trial screening for lung and colorectal cancer. As of October 2020, more than 10,000 people have been recruited for the trial (45). Eligibility criteria were set to be those aged 50–74 years with a 20-pack-year smoking history who are current smokers or who quit within the past 5 years (46). Almost half of lung cancers diagnosed in China are in never-smokers, which suggests that other factors, such as passive smoke, air pollution, low fruit intake, and family history of cancer, play a role (47). Guidelines were updated in 2021 to reflect wider eligibility criteria, including passive smoke exposure, presence of chronic obstructive pulmonary disease (COPD), occupational exposure, and family history of lung cancer (48). Consideration of family history are particularly important to ensure the right population has access to screening, given the high prevalence of lung cancer in non-smokers in China (7). However, there is no centralized implementation body in China; programs are funded and organized by local governments. A simulation model suggests that by 2025, approximately 30 million people will be eligible for lung cancer screening in China based on the existing guidelines (49). No targets for population screening have been identified (see Table 2).

**Table 2:** Summary of the adoption of the large-scale lung cancer screening programs in China.

Region	Year initiated	Screening numbers
Rural People’s Republic of China Screening Program (RuraCSP)	2010	Around 13,000 high-risk individuals were scanned with a detection rate of 1%
Cancer Screening Program in the Urban People’s Republic of China (CanSPUC)	2012	Between 2012 and 2015, 521,302 eligible individuals were identified as high risk; 163,752 (~31%) of them were scanned, and follow-up of the trial is ongoing
People’s Republic of China National Cancer Early Screening Trial screening for lung and colorectal cancer	2019	As of October 2020, more than 10,000 people have been recruited for the trial

Source: CRA analysis.

#### 3.4.2. Adoption

Between 2013 and 2018, screening uptake was recorded at 34, 37, and 48% in Shanxi, Henan, and Zhejiang provinces, respectively (50). However, uptake data from 2018 to 2019 suggests that the participation rate across China nationally is between 6 to 31%, so there is significant variation across the country (51).

#### 3.4.3. Factors affecting adoption

While an LDCT scan costs about US$80 and is covered by insurance, there are concerns associated with the cost burden of follow-up scans, travel, and taking time off work (52).

Distrust between patients and the health system is a societal challenge in China. Surveys seeking the factors affecting patient participation reveal that half of respondents do not trust their physician or hospital and believe that doctors put making money before their patients’ health (52). However, greater awareness about lung cancer motivates participation in screening. Awareness does not necessarily come from conversations with doctors. It is correlated with higher level of education and family history of lung cancer (53). People with an education level of college or above have participation rates of 66%, while those with junior school or lower levels of education have participation rates of 45%. People with a family history of lung cancer have substantially higher participation rates (65%) compared with those that do not have a family history of lung cancer (28%). These studies reveal that health literacy is a key predictor in adherence to cervical, breast, and prostate cancer screening programs (54).

One study identified that medical migration is a challenge to maintaining participation. In China, people have free access to any hospital, which means that selection bias and higher dropout rates are expected when recruiting based on hospital catchment areas, which fluctuate constantly (47).

Finally, disparities in socioeconomic status also correlate with screening uptake: 31.8% in developing areas vs. 37.4% in developed areas as they are less likely to fully utilize healthcare resources and would tend to choose no care over self-care, outpatient care, or in-patient care due to worse health insurance coverage (55).

Other general uptake factors include gender; uptake is higher among women (65%) than men (42%), which tends to be a common observance in other countries, likely due to the attitudes of men toward self-care compared with those of women (53).

Although not identified in studies, it is likely that healthcare infrastructure affects uptake. According to OECD data from 2013, the number of CT scanners per million across five different provinces was at least 50% lower than the OECD average (56). Additionally, there were 220 physicians per 100,000 population, lower than the OECD average of 360 physicians per 100,000 (27).

### 3.5. Croatia

#### 3.5.1. Background

In October 2020, Croatia became the first country in the EU to implement a nationwide lung cancer screening program, which leveraged the political tailwind of the implementation of Croatia’s first national cancer strategy (57). The program was launched for active smokers aged 50–70 years and people who had quit smoking in the past 15 years (58). The working group of the Croatian Thoracic Society and the Section for Thoracic Radiology of the Croatian Society of Radiologists based their recommendation on various world-renowned institutions’ recommendations. The program relies on technologies that improve the accuracy of CT readings and simplify the patient journey by taking advantage of electronic records readily shared in the health system. There are currently 12–16 certified screening facilities in the country, and through the program, Croatia hopes to achieve a screening coverage of 50% of the target population and reduce mortality by 20% in the next 5–10 years (59).

#### 3.5.2. Adoption

As of February 2021, the program had screened over 2,000 people (57). More recent data from 2022 suggest that around 10,000 people have now been screened (57). To date, no formal assessment of the success of the program has been made. However, the literature cites equitable access as an ongoing challenge in the country (60). The committee responsible for creating and monitoring the national program has committed to an annual review.

#### 3.5.3. Factors affecting adoption

Given the relatively recent introduction of the screening program, little review has been conducted. Some of the challenges were anticipated in the guidelines. The guidelines for the national program stress that an important consideration in the design of the lung cancer screening program is the need for engagement from primary care providers, who have the closest contact with the public, to ensure that high-risk patients are being referred correctly so as to increase uptake (61). Unlike other cancers, for which invitations for screening come from the Ministry of Health institute, invitations for lung cancer screening are sent out directly by primary doctors (57). Therefore, good education of general/family doctors is needed to build awareness of the availability of the program (62). In the past 2 years, there have been significant efforts to improve education of physicians, which has likely contributed to the uptick in participation (57).

It is also likely that healthcare resources affect adoption. Croatia has 300 physicians per 100,000 population. This number is lower than the OECD average of 360 physicians per 100,000 inhabitants (63, 64). According to a 2021 study, there were 6 private CT scanners and 6 public CT scanners per 1 million people (65). In 2022, there were 17 CT scanners available to the public—a significant improvement in equipment availability (57).

We did not find literature assessing the impact of out-of-pocket (OOP) costs on lung cancer screening, but screening is unlikely to be significantly financially burdensome because cancer care and treatment is free for all citizens regardless of health insurance status. Moreover, in 2018, out-of-pocket (OOP) payments for all healthcare averaged 10.5% of the total health expenditure in Croatia, well below the EU average of 15.5% (66).

#### 3.5.4. Other cancer screening programs

Among other cancer screening programs, adoption rates have been seen to be relatively low at 22.5% of women for breast cancer and 13.7% of men for prostate cancer, as observed in a 2007 study. The study also reported that utilization of breast, colon, and prostate cancer screening in rural populations is lower than in urban areas across all cancers (breast, 14.5% vs. 27.4%; prostate, 9.6% vs. 16.3%; colon-men, 5.7% vs. 6.3%; colon-women, 3.6% vs. 5.1%, respectively). This suggests that access to healthcare and limitations of infrastructure will be important factors in uptake of cancer screening in Croatia (67).

### 3.6. Canada

#### 3.6.1. Background

**Figure 3:**
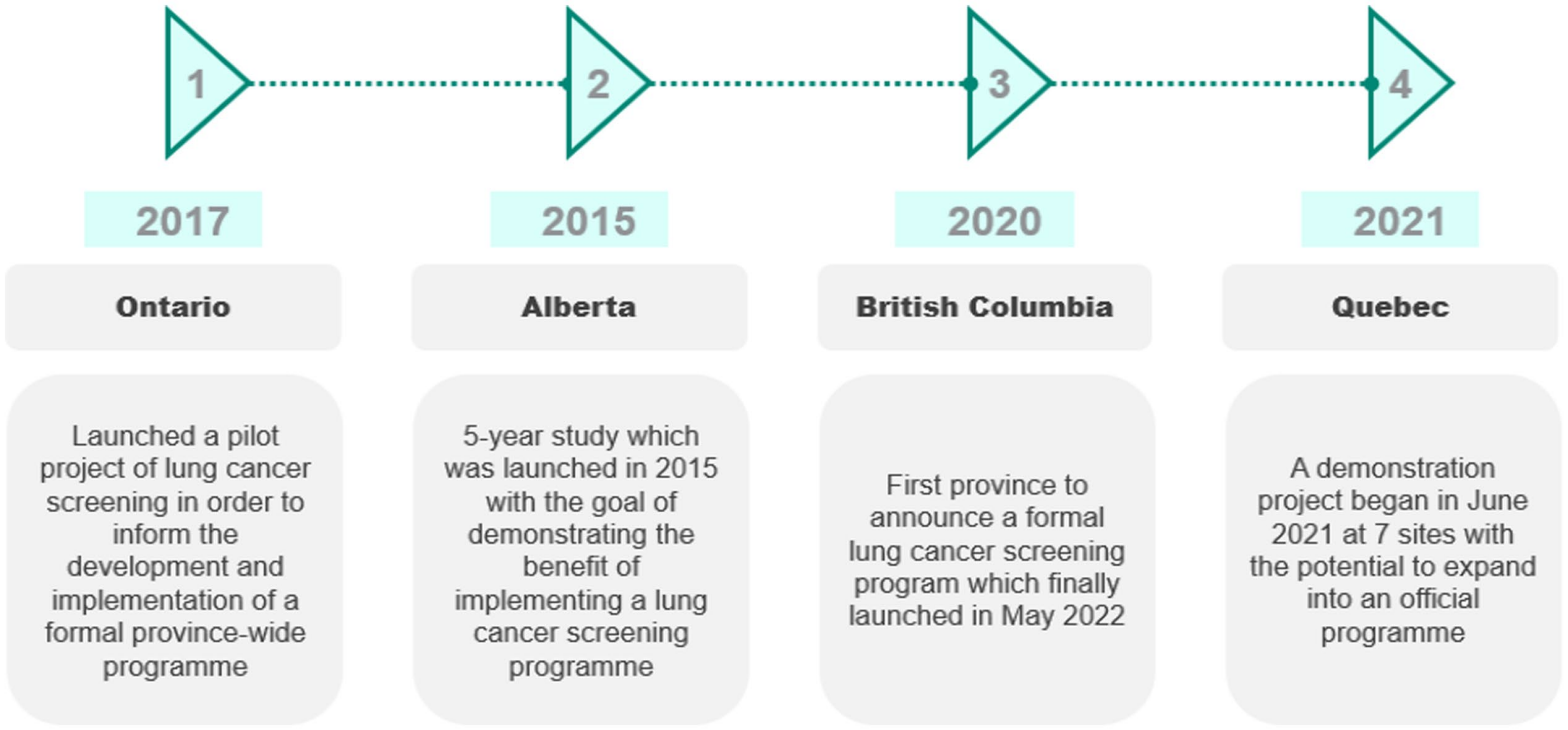
Lung cancer screening across provinces in Canada. Source: CRA analysis.

In 2014, the Canadian Task Force on Preventative Health Care (CTFPHC) reviewed guidelines for lung cancer screening and recommended LDCT screening based on the National Lung Screening Trial (NLST) (68, 69). However, it is the responsibility of the province to establish screening programs (68). Hence, the adoption of lung cancer screening varies by province, summarized in Figure 3.

In 2020, British Columbia (BC) became the first province to announce that it would implement a formal lung cancer screening program (for high-risk individuals aged 55–74 years who are currently or have previously smoked and have a 20-pack-year smoking history) (70). The province of Ontario launched a pilot project of lung cancer screening to inform the development and implementation of a formal province-wide program. The pilot was initiated at three sites in 2017 and ended in 2021 (71). Eligibility criteria for the pilot included being 55–74 years of age and a current or ex-smoker who has smoked daily for at least 20 years (not necessarily consecutively) (72).

In Alberta, the University of Calgary conducted a five-year study beginning in 2015 (73). Eligibility for the program required being aged 50–74 years with a smoking history of at least 30 pack-years. Patients were assessed by their primary care physicians and referred for screening if eligible (74).

A lung cancer screening demonstration project in Quebec began in June 2021 at seven sites for those aged 55–74 years who have smoked for 20 years and quit fewer than 15 years ago. The results of the first 3,000 patients will determine whether the program will be expanded into an official program (75).

#### 3.6.2. Adoption

Data on adoption are limited. In some cases, this is unsurprising—the program in BC has been running for only 3 months. In other cases, the programs have been running longer but there has been little formal assessment.

Looking at the opportunistic programs, a retrospective review of patients undergoing lung cancer screening in Eastern Newfoundland, a region in the province of Newfoundland and Labrador, between 2015 and 2018 found that only 6% of the eligible population is being screened opportunistically in this province, which has no formal program (68). An audit of the opportunistic program suggests that rural communities have less access to health services (the rural population is disproportionately higher in Eastern Newfoundland, at 34.3%, compared to Canada as a whole, 18.9%) (68).

#### 3.6.3. Factors affecting adoption

Few studies have looked at the factors affecting adoption. According to the limited literature, they have concluded, on the basis of the Ontario lung screening pilot, that women have been more likely to participate than men. They related this to awareness, concluding that screening programs may benefit from targeted recruitment and marketing efforts toward screen-eligible men in addition to broader awareness campaigns (76).

Although OECD data from 2019 showed that Canada had 14.6 CT scanners per 1 million population, much less than the OECD average of 25.9 per 1 million population, literature suggesting that infrastructure is a barrier to adoption in Canada is limited (15). Given the funding structure of Canadians’ healthcare, financial considerations are unlikely to play an important part as healthcare services are covered by public financing. We cannot find the cost of lung cancer screening, but other tumor-type screening programs, such as for breast cancer, are free at the point of use.

#### 3.6.4. Other cancer screening programs

The first breast cancer screening program in Canada was implemented in 1988 in British Columbia; it was followed by the implementation of programs in 11 more provinces between 1990 and 2008 (77). Adoption rates have remained stable, at approximately 54%, since 2011 (77).

A 2008 survey looking at screening rates in breast, cervical, and colorectal cancer found a difference in uptake between urban (22%) and rural remote (18%) populations (78). Among those yet to be screened for breast cancer, a large majority tend to be from groups that have access to the fewest resources, be the most isolated, and experience poorer outcomes (78). A survey in 2008 showed that immigrants who had lived in Canada fewer than 10 years had a lower uptake of mammography screening (40%) compared with those who had lived in Canada for more than 10 years (70%) (78). Many of these barriers are likely to also exist in lung cancer because those at higher risk of the disease tend to fall into lower income groups and often have less access to screening.

### 3.7. Poland

#### 3.7.1. Background

In 2008, four early-detection pilot studies were implemented by thoracic surgeons in Szczecin, Gdansk, Poznan, and Warsaw (79). The programs are summarized in Table 3. These pilot programs had different durations. By 2018, a total of 50,000 high-risk people had been screened (80). The inclusion criteria for all the sites were similar and included being aged 50–70 years or being at least 75 years of age with a smoking history of 20 pack-years (79).

**Table 3:** Features of lung cancer screening pilots across regions in Poland.

Region	Features of pilot programs
Szczecin	The program was fully financed by regional government funds
Gdańsk	The program was accompanied by complementary biomarker projects and funded by grants from Polish scientific institutions that were the equivalent of 6 million Euros
Poznań	The program was fully financed by regional government funds
Warsaw	This is the region in the country with the lowest rates of operable NSCLC at diagnosis (lowest resection rates) and, therefore, greatest need for early and timely diagnosis; the program was financed by the national Ministry of Health

Source: CRA analysis.

The final results from the NELSON trial prompted the development of a national pilot program in 2020 for 3 years across the entire country, divided into six main regions, with a commitment to set up the formal national program in 2023 (see Table 3) (60, 81). Inclusion criteria for the national program are being 55–74 years of age and having a smoking history of 20 pack-years (active smoking or having quit no longer than 15 years ago) or being aged 50–55 years of age and having the same smoking history criteria and an additional risk factor, such as exposure to toxic compounds, previous cancer history, or diagnosis of chronic obstructive pulmonary disease (COPD) or idiopathic pulmonary fibrosis (IPF) (82).

#### 3.7.2. Adoption

To date, no assessment on the uptake or success of the program has been identified.

#### 3.7.3. Factors affecting adoption

Interest in participation in the first 2 years of the Gdansk pilot was high, exceeding capabilities, but it waned substantially in 2016–2018 (79). This suggests that the recruitment strategy is key in improving and maintaining high uptake following the initial attention around the program (83). Closely cooperating with family doctors was seen as vital in the recruitment strategy to ensure that high-risk participants were referred for screening (83).

Workforce capacity is also seen as important, with an experienced team being another contributing factor. Notably, the screening experience gained by the team during the first pilot program resulted in reduced false positives, unnecessary diagnostics, and surgeries. Both programs had a mortality rate of zero and a low complication rate (79).

These lessons learnt were considered in the design of the national program to stimulate uptake. A centralized approach is used to ensure efficient use of the workforce with one leading center, appointed by the Ministry of Health, working directly with regional screening centers. Poland recognized the need for direct contact with the target population in the recruitment strategy. Each regional screening center collaborates with around 40 primary care centers, involving a total 600 primary care centers across the country. Each of the centers is provided with leaflets and educational materials to share with potential screening candidates (60).

It is unlikely that financial considerations play a direct role. Healthcare in Poland is free for all citizens and provided through the publicly funded healthcare system, as is the official lung cancer screening program (79).

However, it is likely—though this is not identified in the literature—that healthcare infrastructure is a determinant. Data from 2019 show that Poland had 18.2 CT scanners per 1 million people, which is significantly lower than the OECD average of 25.9 (15). Data from 2019 showed that Poland had 240 physicians per 100,000 population, lower than the OECD average of 360 physicians per 100,000 population (15).

#### 3.7.4. Other cancer screening programs

Assessment of other cancer screening programs shows generally poor uptake, suggesting that inherent challenges to adoption of screening exist in the healthcare system. Cervical cancer screening was introduced in 2006, and between 2007 and 2013, uptake was around 10–13% (84). Promoting cervical cancer screening in primary care settings has been thought to be key to improving screening uptake (84). A systematic review assessing breast and cervical screening programs in Poland suggested that a higher level of education and employment correlates with greater uptake (85).

## 4. Discussion

**Figure 4:**
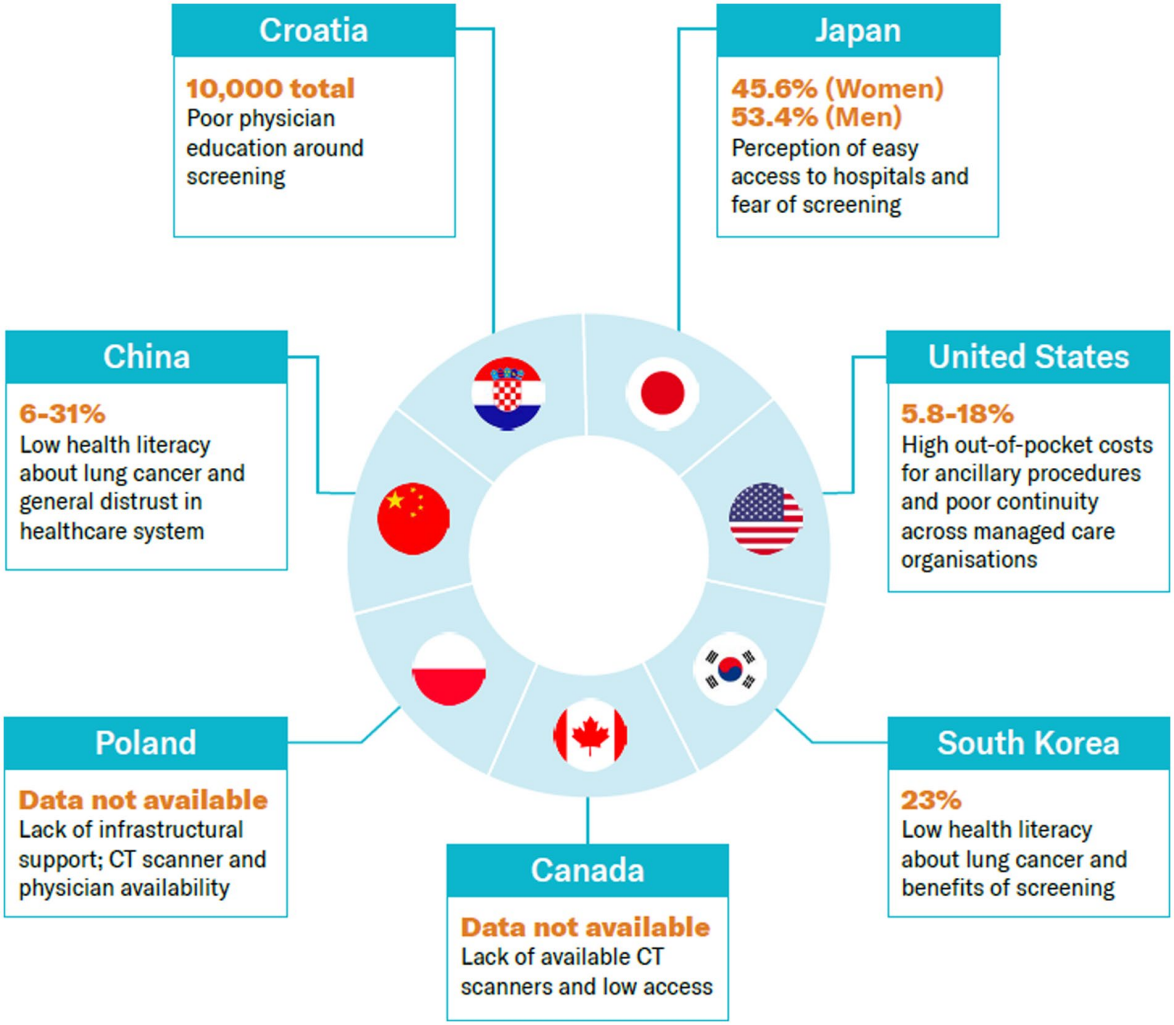
Adoption rates across countries and key drivers. Source: CRA analysis.

Our research suggests that we can stratify factors affecting adoption of lung cancer screening into two groups: health system readiness and individual cancer literacy. Within each area are specific themes driving uptake of lung cancer screening. See Figure 4. Definitions of the factors are found in Supplementary material.

### 4.1. Health system readiness

#### 4.1.1. Political

National targets in lung cancer outcomes or screening have not been consistently identified in the countries studied. The establishment of national goals in Croatia, the first country in the EU to implement a formal program, was influential in driving the implementation and design of the lung cancer screening program. Croatia hopes to reduce lung cancer mortality by 20% in the next 5–10 years through the screening program and achieve a screening coverage of 50% of the target population. This ultimately shaped the design of the program to ensure successful uptake: the program included direct outreach by primary care providers, who have the closest contact with the public, to ensure that high-risk patients are being referred correctly to increase uptake, and education of general/family doctors to build awareness of the availability of the program. This experience suggests that political prioritization of lung cancer is pivotal in driving momentum in the service, and having a clearly communicated goal allows the public and health care professionals to understand the vision and move in that direction.

#### 4.1.2. Financial

Comparing states that cover lung cancer screening to those that do not revealed that patients with insurance had modestly higher screening rates (15.7% vs. 9.3%). However, other studies suggest that the right population needs to have access to reimbursed screening services. A study of Medicaid/Medicare coverage in the US demonstrated that 87.1% of eligible patients were not being screened, despite having insurance coverage. Inability to access reimbursed services has been cited as a key reason for low screening rates. For example, the American College of Radiology identified that as of February 2021, Medicare administrative contractors are still not correctly adhering to Medicare guidelines and continue to deny claims billed by independent diagnostic testing facilities. Furthermore, a nationwide study in the US (2016) across Federally Qualified Health Centers that provide care to low-income communities reported lack of insurance, challenges in obtaining prior authorization, and coverage denials in 72, 58, and 30% of respondents, respectively.

In countries such as Japan, South Korea, Croatia, and Poland where a public financing system is in place, out-of-pocket (OOP) payments for screening and downstream diagnostic tests and treatment are manageable but challenges in adoption remain due to the potential costs of ancillary activities. Patients must cover the costs of transportation and/or taking sick leave to attend the appointment, which can be particularly challenging for people in low-income settings.

#### 4.1.3. Infrastructural

The number of CT scans and certified facilities, as well as appropriately trained workforce for the screening programs, affects broad uptake of LDCT screening. Despite limited supportive evidence from the literature in countries such as China, Croatia, and Poland, where the number of CT scanners is significantly lower than the OECD average, the healthcare infrastructure probably affects uptake. By contrast, this probably is not the case in Japan, the US, South Korea, and Canada, where the availability of CT, MRI, and PET scanners and the number of physicians relative to the population are high.

As shown by the Association of American Medical Colleges, the northeast has the highest ratio of physicians per 100,000 population and the most certified screening facilities; these facts correlate with the highest uptake rate for lung cancer screening in the US. In Canada, breast cancer screening adoption varied across provinces, and those with a lower number of physicians *per capita* tended to have a high percentage of patients without a regular doctor and a lower screening uptake. Poland considered the efficient use of the available workforce when designing its program.

Physician awareness of the benefits of lung cancer screening affects referral to screening services. Qualitative studies in the US suggest that poor experiences with GPs during consultation prevent further participation in lung cancer screening programs. Surveyed participants reported invitations from GPs were opportunistic and unaccompanied by a clear explanation of the procedure; only 42% of HCPs were able to accurately identify high-risk patients. This suggests that greater physician education efforts are needed, especially given the key role GPs play in making referrals. In the design of the lung cancer program in Croatia, guidelines stressed the need for engagement by primary care providers, who have the closest contact with the public, to ensure that high-risk patients are being referred correctly so as to increase uptake. Similarly, in Poland, governments recognized the need for direct contact with the target population to be part of the recruitment strategy; each regional screening center collaborates with around 40 primary care centers (a total of 600 primary care centers across the country), and each center is provided with leaflets and educational materials to share with potential screening candidates. The challenge is further exacerbated for nonsmokers where no guidelines for this population exist.

Finally, access to screening is critical, especially for low-income households, as demonstrated by adoption data comparing rural and urban areas in Canada, China, and Croatia. A public opinion survey on the National Lung Cancer Screening Programme in South Korea suggested that lower-income households have a strong preference for accessibility of the screening units over quality (accessibility being defined in terms of travel distance and quality being defined as the screening occurring in a large referral hospital vs. a small hospital). Lack of cultural awareness and language diversity within healthcare providers can drive low participation by lower socioeconomic groups. A study looking at uptake of cancer screening among immigrants who had lived in Canada less than 10 years revealed that such persons had a lower uptake of mammography screening, at 40%, compared with those who had lived in Canada for more than 10 years, at 70%.

### 4.2. Individual readiness

#### 4.2.1. Cancer literacy

A person’s ability to discern health information related to lung cancer screening and assess their own individual risk is critical for improved screening participation. In Japan, a 2022 study assessing lung cancer screening rates and related factors in the child-rearing generation suggested that those with higher education, i.e., a university or graduate school education, had a greater desire to be aware of their health status and therefore more participation in screening. While studies of public awareness of lung cancer screening are limited in Poland, we can draw conclusions about its potential impact on adoption from other cancer screening programs. A systematic review assessing breast and cervical screening programs in Poland suggested that a higher level of education and employment correlates with a higher uptake rate. A similar finding was identified in China, where those with a college-level education and above had higher participation rates. It is likely that more education results in greater health literacy and better understanding of health-related information, which are needed in decision-making. In addition, public empowerment to understand one’s health condition also differs by gender likely due to the attitudes of men toward self-care compared with those of women.

Conversely, a lack of understanding of the effectiveness and benefits of LDCT screening among the public hinders adoption, as demonstrated by studies in Japan, the US, China, and Canada. In Japan, respondents in public opinion surveys cite “lack of time,” “confidence in health condition,” and “ease of access to hospitals if and when needed” as key reasons for nonparticipation; this suggests a lack of understanding of the risks of lung cancer and the importance of early detection. Similarly, the latest public survey in 2018 conducted by the National Cancer Centre in South Korea suggests that the key reason for low uptake is low public awareness of the necessity of cancer screening. In the survey, 42.5% of respondents answered “I am still healthy” as the key reason for nonparticipation in cancer screening. Generally, there is poor awareness of the benefits of screening and the need for early diagnosis.

In addition, it’s worth nothing that people with a family member who has had cancer have a better understanding of cancer and are more receptive to screening, as identified in Japan and some other countries. In addition, studies in Canada and South Korea suggest that women are more likely to participate in screening, given their greater desire to be aware of their health status compared to men. All of this suggests that people who are more informed about the disease and proactive in seeking health care are more inclined to participate in screening programs.

#### 4.2.2. Cultural

Fear of cancer or cancer screening prevents participation. A study of Korean men found they had a fear of radiation exposure and perceptions that the LDCT test is painful. The 2015 Korean National Cancer Screening Survey sampled 1,730 men aged 40–74 years and found that this perception was more prominent in high-risk vs. average-risk groups—58 and 49%, respectively. Another review identified that not only was there a fear of pain from the tests but generally fear of undergoing unnecessary radiation exposture, overdiagnosis and false positives would do more harm (86).

Moreover, stigma from being blamed because of negative societal perceptions of tobacco use has been identified as a particular challenge in lung cancer screening uptake in the US. However, positive experiences in local pilots have suggested that direct outreach from primary care providers, such as pharmacists and GPs/family doctors, breaks down the barriers of stigma and improves adherence, as demonstrated in pilot programs in Poland and Croatia.

General distrust of the healthcare system impairs adoption, as demonstrated in the US. Similarly, in China, 50% of respondents in a study assessing uptake in the middle-aged population claimed that they do not trust their physician or hospital; patients perceive that doctors put making money before their patients’ health and therefore are skeptical when doctors recommend screening.

The five themes outlined above are distilled from publicly available literature predominantly focused on middle- and higher-income countries, where lung cancer screening is more advanced. Our research does not rank the importance of these factors, as each carries different weighting across the types of health systems and no single factor can be viewed in isolation as the universal sole driver of participation across all countries. Instead, these five themes are often cited in the literature as barriers to or enablers of uptake of lung cancer screening particularly and are a starting point for discussion of the design of successful programs. Given the small number of countries studied and the limitations of the available literature, these countries may not be representative of the lung cancer screening experience in all settings, particularly to draw conclusions on the geographical regions or patient sub-groups. and in lower- and middle-income countries where health systems are often under-resourced. Given the risk profile and genetic landscapes and high prevalence of lung cancer in nonsmokers in Asia, further research is needed to understand differences in the barriers across geography and patient groups to tailor strategies.

The objective of this paper is to understand the factors that affect adoption of national lung cancer screening programs. Drawing on the available information on current lung cancer screening programs; past experiences in pilot programs and other cancer screening programs; and validation by experts representing perspectives from policy, payers, patients, and the private sector, we concluded that five key themes play a role in the uptake of lung cancer screening programs:

#### 4.2.3. Health system readiness

- Political will in setting and meeting national lung cancer objectives can be the driving force in directing time and resources toward ensuring that lung cancer screening programs are successful.
- The financial burden on the public of accessing lung cancer screening and post-screening services is a significant barrier to participation.
- Healthcare infrastructure to support screening services, including medical equipment and trained personnel in the right settings, accessible to those who are at greater risk of lung cancer affects screening uptake.

#### 4.2.4. Individual readiness

- Empowerment to understand one’s health which may differ by gender and the awareness of and ability to discern health-related information about improving lung cancer outcomes are critical to motivation to participate in screening.
- Fear of lung cancer and stigma, as well as distrust of the health system, impede the adoption of lung cancer screening, and breaking down these societal factors is critical for unlocking participation.

Our research aims to serve as a resource for lung cancer screening patient groups and policy makers that will help them design a successful program and direct resources to supportive systems. Further research may be needed to understand how increasing adoption of lung cancer screening may affect access to cancer care across income levels and have implications for achieving health equity. For the best health outcomes for all, future design of screening programs will need to balance program effectiveness, cost-efficiency, and fairness.

## Author contributions

CP: Conceptualization, Project administration, Writing – original draft. TW: Conceptualization, Supervision, Writing – review & editing. IS: Writing – original draft. AR: Supervision, Writing – review & editing. MY: Supervision, Writing – review & editing.
