# Supplementary material for: Why is the screening rate in lung cancer still low? A seven-country analysis of the factors affecting adoption
Source: Front Public Health. 2023 Nov 9;11:1264342. doi: 10.3389/fpubh.2023.1264342 (PMC10666168; doi:10.3389/fpubh.2023.1264342)
Supplement: Supplementary file 1 [file Table_1.docx]

**Supplementary: Definitions of the factors affecting adoption of lung cancer screening**

**Health system readiness**

**Political**

Political prioritization of lung cancer or lung cancer screening through setting of national targets

**Financial**

Financial incentives and cost sharing for screening and related doctor visits, testing, and treatment if needed; ancillary costs include expenses for travel and time off work

**Infrastructural**

Capacity of the underlying infrastructure to support the provision of lung cancer screening, including availability of CT scanners and trained radiologists; high awareness among physicians of the benefits and risks of screening and existing screening programmes; and broader social determinants such as socioeconomic status/health inequities that implicate access to the service

**Individual readiness**

**Cancer literacy**

Individuals’ ability to discern information; gain knowledge about lung cancer, their risk level, and the benefits of screening and timely diagnosis; and stay informed about lung cancer screening as a service

**Cultural**

Individuals’ views about lung cancer and screening, which are affected by factors such as stigma associated with tobacco use, trust/distrust in the health system, and screening promotion by celebrities

Source: CRA analysis
